# Preliminary Assessment of Parmigiano Reggiano Authenticity by Handheld Raman Spectroscopy

**DOI:** 10.3390/foods9111563

**Published:** 2020-10-28

**Authors:** Mario Li Vigni, Caterina Durante, Sara Michelini, Marco Nocetti, Marina Cocchi

**Affiliations:** 1 Dipartimento Scienze Chimiche e Geologiche, Università di Modena e Reggio Emilia, Via Campi 103, 41125 Modena, Italy; emmellevi@gmail.com (M.L.V.); durante.caterina@gmail.com (C.D.); 2 Consorzio Parmigiano Reggiano, Via Kennedy 18, 42124 Reggio Emilia, Italy; michelini@parmigianoreggiano.it (S.M.); nocetti@parmigianoreggiano.it (M.N.)

**Keywords:** Parmigiano Reggiano, grated cheese, handheld Raman, SIMCA, PLS, authenticity, chemometrics

## Abstract

Raman spectroscopy, and handheld spectrometers in particular, are gaining increasing attention in food quality control as a fast, portable, non-destructive technique. Furthermore, this technology also allows for measuring the intact sample through the packaging and, with respect to near infrared spectroscopy, it is not affected by the water content of the samples. In this work, we evaluate the potential of the methodology to model, by multivariate data analysis, the authenticity of Parmigiano Reggiano cheese, which is one of the most well-known and appreciated hard cheeses worldwide, with protected denomination of origin (PDO). On the other hand, it is also highly subject to counterfeiting. In particular, it is critical to assess the authenticity of grated cheese, to which, under strictly specified conditions, the PDO is extended. To this aim, it would be highly valuable to develop an authenticity model based on a fast, non-destructive technique. In this work, we present preliminary results obtained by a handheld Raman spectrometer and class-modeling (Soft Independent Modeling of Class Analogy, SIMCA), which are extremely promising, showing sensitivity and specificity of 100% for the test set. Moreover, another salient issue, namely the percentage of rind in grated cheese, was addressed by developing a multivariate calibration model based on Raman spectra. It was possible to obtain a prediction error around 5%, with 18% being the maximum content allowed by the production protocol.

## 1. Introduction

Developing objective analytical methodologies for food authentication has been one of the main issues since the introduction of the European Community regulation on quality labels [1]. In particular, the protected denomination of origin (PDO) is a quality marker designed to protect and valorize traditional food, recognizing the link between the intrinsic characteristics of the product and the geographical area (*terroir*) as well as the skillfulness of producers (*savoir faire*). The production protocol accompanying the certification specifies all the needed requirements for the use of the denomination, and authenticity assessment has to cover all aspects herein defined. Authenticity thus encompasses several characteristics of a foodstuff and aims at defining its uniqueness or identity. In this respect, a holistic approach to food characterization, based on so-called fingerprint techniques, is emerging as most promising [2,3], and fast, non-destructive spectroscopic techniques, such as mid- (MIR) and near-infrared (NIR), Raman, nuclear magnetic resonance (NMR) aided by chemometrics modelling are used in wide-ranging applications [4,5,6,7,8], especially because they are suitable for a wide screening campaign and, in the case of NIR and Raman, for in situ analysis thanks to the development of handheld instruments.

Raman spectroscopy may be seen as complementary with respect to MIR and NIR in food analysis [4,8,9], offering some advantages such as: an almost absent interference from water, which makes the analysis of aqueous solutions or samples with a higher water percentage easier and more effective; inorganic components are more easily analyzed, and samples can be directly analyzed through glass or polymer packaging. On the other hand, there also some disadvantages due to the interference from fluorescence of impurities or of the sample itself, the intensity of laser radiation which may result in too strong sample heating, sensitivity to sample temperature fluctuations, and the reduced size of the sampled area. However, instrumentation is constantly improving—in particular, handheld devices are particularly appealing, allowing in-field analysis and being easy to handle.

Several successful applications have been reported in the analysis of spirits [10], meat [11], edible oils [12] and cheese [13,14,15,16]. Here, we present a feasibility study to assess Parmigiano Reggiano cheese by using a handheld Raman device. To the best of our knowledge, this is the first time Raman spectroscopy has been used to characterize Parmigiano Reggiano cheese.

Parmigiano Reggiano is one of the most well-known and appreciated hard cheeses worldwide, and is manufactured from raw and unheated bovine milk in a restricted area in Northern Italy. It gained the PDO denomination in 2012 (European Regulation No. 1151/2012) and must meet specific technological, organoleptically and compositional requirements as defined in the Specifications of Parmigiano Reggiano Cheese protocol [17]. On the other hand, it is also highly subject to counterfeiting. In particular, it is critical to assess the authenticity of grated cheese, to which, under strictly specified conditions, the PDO is extended. Grated Parmigiano Reggiano should be obtained from the cheese wheel and be placed under the constraints of packaging immediately after grating and in the production area of origin. In addition, several characteristic parameters should be met, such as a minimum 12 months of ripening, a fat content no less than 32% in proportion to dry matter, moisture between 25% and 35% and rind content not higher than 18% (*w*/*w*).

In this work, we evaluate the potential use of a handheld Raman device, which allows for acquiring spectra directly through packaging. This methodology would allow fast, non-destructive and in situ screening of potentially every package, which is of great interest both for internal control of compliance at the Consortium and for fraud monitoring in the marketplace.

This feasibility study aims at evaluating whether the acquired Raman spectra could be used as a fingerprint of the product to build multivariate models for: (i) recognizing the authenticity of grated Protected Denomination of Origin (PDO) Parmigiano Reggiano cheese, in order to prevent fraud and misleading commercial practices (class modeling), and (ii) verifying the compliance of authentic grated Parmigiano cheese with respect to the maximum allowed rind content percentage (multivariate calibration).

With respect to the first issue, the current methodology is based on the assessment of stable light isotopes and mineral profiles [18,19], which Grana Padano and Parmigiano Reggiano Cheese Consortium officially adopted in 2011 (EU Regulation 1151/2012). This is a destructive and quite costly methodology. In recent years, on the side of non-destructive fingerprint techniques, feasibility studies with NIR, MIR [20,21], NMR [22], and nanowire gas sensor devices have been reported [23].

With respect to the limit of 18% of rind in grated cheese, the current methodology is based on capillary electrophoresis, which was one of the first technique used to determine the rind content in grated cheese. The method is based on the determination of the different patterns of casein degradation in rind and pulp [24]. This procedure is quite expensive and time-consuming, and the assignment is based on the determination of the ratio among two casein sub-fractions, establishing a threshold value below which the rind content is inferior to 18% [25].

More recently, feasibility studies based on nondestructive techniques, such as gas sensors [26], NIR, MIR [20,21,27] and NIR imaging spectroscopy [28], were reported, and the lowest prediction error was obtained by NIR imaging. Nonetheless, none of these were applied on intact packaging.

## 2. Materials and Methods

### 2.1. Sampling

Eighty authentic PDO Parmigiano Reggiano cheese samples (PR) from two different production years and different seasoning, ranging from 11 to 15 months, were provided by the Parmigiano Reggiano Cheese Consortium. These included 28 samples with a rind content between 8% and 18% (compliant samples), and 52 samples with a rind content higher than 18% (up to 42%, non-compliant). The samples were derived from certified whole cheese wheels; rind and cheese pulp were grated separately, and the nominal rind content was added by weighing (balance sensitivity 0.01 g). Cheese rind was obtained by cutting the first top 6 mm from the whole cheese wheel, while cheese pulp was obtained from the internal part at least 2.1 cm depth from the top. The grated samples were then packaged following the procedure described in the standard production protocol specification [17]. In this preliminary study, bags of the same material, i.e., polyethylene/polyamide not printed, were employed, both for PR and not-PR samples, while in current practice the bags’ material may differ.

Additionally, 20 cheese samples, unknown to us, were provided by the Parmigiano Reggiano Cheese Consortium at a second time point—10 samples were authentic PR with a varying percentage of rind (prepared as described previously) and 10 samples were not-PR cheese samples from competitors, of different provenance. In Table 1 are reported the number of samples analyzed per each category, the year of production and the date of preparation of the grated mixtures. The seasoning of PR samples varied within the range of 11 to 15 months.

### 2.2. Raman Spectra

Spectra were acquired directly on packaged grated samples (sealed plastic bag) with a handheld Raman spectrometer. The instrument used is a Metrohm MIRA-M1 equipped with a 785 nm Laser and implementing Orbital Raster Scan (ORS) technology [29,30]. This modality allows for increasing the interrogation area on a sample while maintaining high spectral resolution. The spectra were acquired in the spectral range 400–2300 cm⁻^1^, and the acquisition time was set to 4.5 s, after some preliminary trials, because at shorter times dependence of spectral intensity on acquisition time was observed, and a higher dispersion of replicated spectra in an exploratory principal component analysis.

In order to get a representative spectrum, for each packaged sample, five points were sampled (located on proximity of the four corners and in the middle of the bag) and the five collected spectra averaged. For each sample, this average spectrum was used in the chemometric analysis.

The samples were measured on two different days (May 2016): the samples coded PR 1st in Table 1 were measured on the first day, and samples coded PR 2nd, PR-unknown and not-PR were measured on the second day. For each set of acquisitions, a sample was randomly selected and acquired several times during the measuring session to get a rough estimate of spectra reproducibility; in particular, one sample of the PR 1st set was replicated four times and one sample of the PR 2nd set was replicated six times.

### 2.3. Spectral Preprocessing

**Figure 1:**
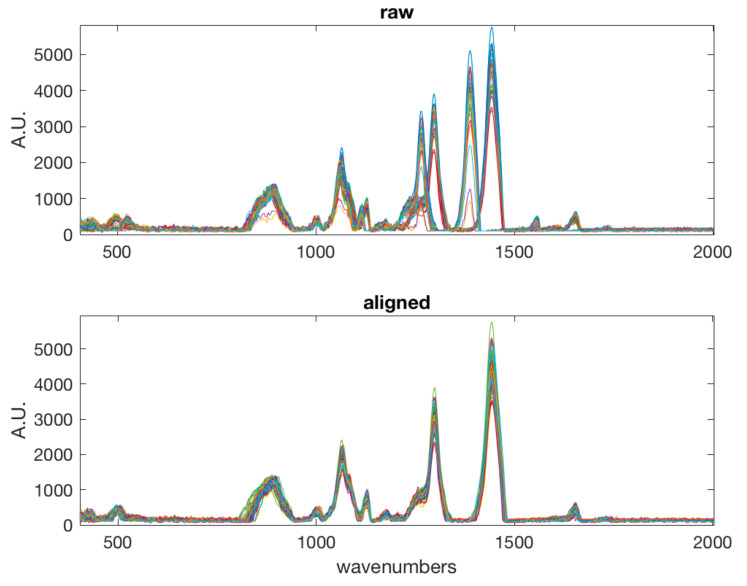
Raw (**top**) and aligned spectra (**bottom**).

**Figure 2:**
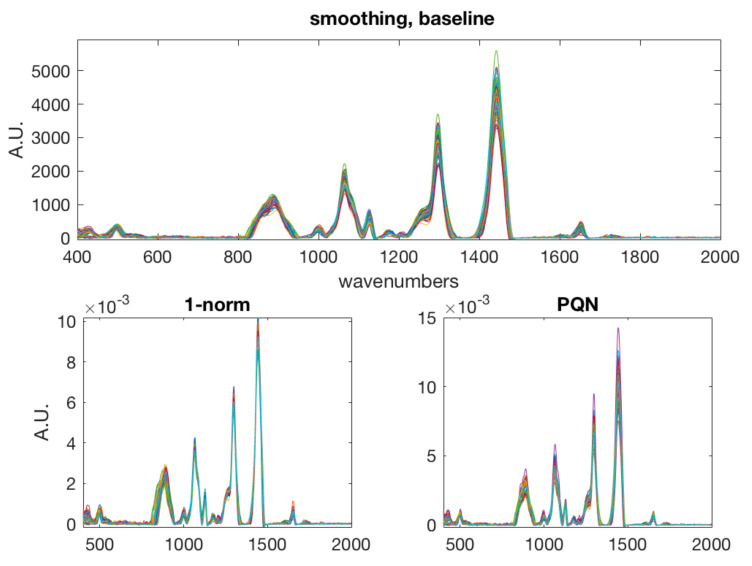
Aligned spectra after smoothing and baseline correction (**top**), normalization to unit area (**bottom left**) and probabilistic quotient normalization (**bottom right**).

There are several contributions introducing variability in a Raman spectrum which may adversely affect the analytical results, particularly when multivariate data analysis is applied. In fact, these introduce undesired sources of variability, such as noise, scattering, baseline drift. These can be due to instrumental fluctuations and slight variation in the distance between samples and optics, and the presence of fluorescence. In addition, variation in sample state, e.g., linked to temperature, may introduce moderate misalignment. Thus, it is necessary to apply spectral preprocessing. In this work, the following spectral preprocessing steps were applied in sequence: smoothing (Savitzky-Golay filter width: 21 points, 2nd order polynomial); alignment by correlation optimized warping [31], which was needed to compensate the shift observed between the two days of measurement; baseline correction by weighted least squares [32] and normalization. In the case of the classification model, simple normalization to unit area was effective, while in the case of multivariate calibration of rind percentage, normalization by using probabilistic quotient normalization [33] was more effective, producing a lower prediction error in the calibration model. Figure 1 and Figure 2 show the raw spectra and the spectra after the various preprocessing steps.

### 2.4. Classification Analysis

The authenticity model was developed by using a class modeling technique, i.e., Soft Independent Modeling of Class Analogy (SIMCA) [34,35], since we need to develop a one-class model, namely to assess if a grated cheese sample is or not obtained by authentic wheels of Parmigiano Reggiano cheese (PR).

#### 2.4.1. Datasets

For authenticity assessment, we considered all the available authentic Parmigiano Reggiano cheese samples (independently of the rind content), namely samples coded PR 1st and PR 2nd in Table 1, which amount to 80 samples. The calibration dataset included the preprocessed spectra of all PR samples plus 10 replicates (as detailed in Section 2.2).

The origin of the twenty additional samples reported in Table 1 as unknown-PR and not-PR, at the time of analysis, was unknown to us and thus constituted an external test set (20 samples plus 2 replicates) to assess the predictive performance of the SIMCA model.

At a second time point, when the origin of these samples was communicated to us, for comparative purposes a second SIMCA model was computed, by using five of the not-PR samples to estimate the rejection rate of the model in the calibration phase (as detailed in next section). The 10 unknown-PR (plus one replicate) and the 5 not-PR (plus two replicates) were used as a test set.

#### 2.4.2. Soft Independent Modeling of Class Analogy (SIMCA)

SIMCA is based on building a disjoint principal component analysis (PCA) model for each class (if more than one is modeled)—this is assumed to describe at best the similarity of the samples belonging to each given class, or, in other words, their uniqueness. Data preprocessing, e.g., centering, is done separately for each class, as well as the number of components is established independently for each class. The classification rule is defined on the basis of distance of the sample to the class model and two distances are defined: (i) the scores distance (*SD*), which indicates how far a sample is from the training objects of the class in the principal component (PC) space (distance in model space), and (ii) the orthogonal distance (*OD*), which measures the distance of a sample to the PC space of the class (distance from model space) and is given by the sum of squared residuals. However, there are different implementations of SIMCA which differ by the way *SD* is defined, how *SD* and *OD* are combined to obtain an overall distance, *D*, from the class model and the reference statistics (or robust estimation) used to define acceptance limits for *SD*, *OD* or *D*.

Here we used the implementation called *alternative*-SIMCA [35], which uses as a classification rule the reduced distance:

$$D_{red}=\;\sqrt{{\left(\frac{Q}{Q_{lim}}\right)}^{2}+{\left(\frac{T^{2}}{T^{2}{}_{lim}}\right)}^{2}\;}\leq \;\sqrt{2}$$

where *Q* is the sum of squared residuals (*OD*), $T^{2}$ is the Mahalanobis distance from the origin in PCA scores space (*SD*), and $Q_{lim}$ and $T^{2}{}_{lim}$ are derived, at a specified significance level, by the χ^2^ [36] and Hoteling-$T^{2}$ distributions [37], respectively.

The classification performance of a SIMCA model can be evaluated in terms of:

(i) sensitivity, which is defined as the number of samples correctly identified as belonging to the modelled class, or true positive rate, i.e., TP/N_samples_in_target_class_;

(ii) specificity, which is defined as the number of samples not belonging to the modelled class correctly rejected, or true negative rate, i.e., TN/N_sample_not_in target_class_;

(iii) efficiency, which is defined as the geometric mean of sensitivity and specificity.

There are two main parameters which need to be set in SIMCA:

(1) the model complexity, i.e., the number of components optimal to describe the class model. This can be estimated in cross-validation, by maximizing the sensitivity, e.g., one-class case, or when samples from non-target class are available, by maximizing efficiency or any suitable compromise among sensitivity and specificity values;

(2) the class boundary, which allows for defining the class acceptance area. In alternative-SIMCA, this is usually set by choosing a priori a certain significance level, e.g., the 95th percentile of the references *SD* and *OD* distributions (α = 0.05).

However, there are also approaches where the two parameters model’s complexity and significance level are not tuned independently—in particular, we tested:

(i) a recently proposed one [38], which simultaneously optimizes the significance level and the number of components by maximizing the area under the cross-validated receiver operating characteristic (ROC) curve. ROC is obtained by plotting (1-specificity) vs. sensitivity, by varying overall distance (*D*) threshold and number of PCs. We will refer to this approach as ROC-SIMCA, now on in the following text;

(ii) the Data Driven SIMCA (DDSIMCA) approach [39], which allows us to calculate the errors of misclassification theoretically [40], and where significance level and model complexity are set sequentially: first the significance level for the combined class distance distribution is set a priori, e.g., α = 0.05, then the number of components is (manually) tuned to obtain a sensitivity (in calibration) which will correspond to the nominal α, e.g., if α is set equal to 0.05, a sensitivity of (or most close to) 95% has to be achieved.

In this study, at the beginning, only the PR 1st and PR 2nd samples (calibration set) were available. In this case, the number of principal components (PCs) was estimated according to two different criteria: the minimum of root mean squares error in cross-validation, RMSECV (venetian blind, ten splits), and the maximum sensitivity estimated in cross validation. For comparative purposes, the DDSIMCA approach was also applied.

Once the origin of unknown samples (reported in Table 1) was communicated to us, we tested how the number of PCs used to build the SIMCA class model would change by using five (selected by duplex algorithm [41]) not-PR samples to estimate the class specificity parameter. In this case, as the classification criterion to select the number of PCs, the maximum efficiency estimated in cross-validation was used. For comparative purposes, the ROC-SIMCA approach was also applied.

### 2.5. Multivariate Calibration (PLS) of Rind Content

Partial least squares regression (PLS) [42] was used to derive a calibration model for the rind content. In order to stay close to the situation that will be encountered in routine application, a first model was developed by using PR 1st samples, collected in a different period (Table 1) and measured on a different day, as calibration set. The PR 2nd plus the ten unknown-PR samples were estimated as a test set. As will be discussed later, this model showed systematic error in prediction, thus a model including a small subset of samples from the PR 2nd set, i.e., six samples selected by the duplex algorithm, was also developed. Spectral data were processed as described in Section 2.3 and mean centered prior to PLS.

The number of PLS components was selected according to first minimum in RMSECV (random subsets, 8 splits, 20 iterations).

### 2.6. Software

The whole data analysis process was carried out on MATLAB 2018b (Mathworks, Natick, MA, USA). PLS-Toolbox v.8.7 (Eigenvector Inc., Manson, WA USA) was used for spectral preprocessing and PLS modeling. SIMCA analysis was conducted by using a MATLAB routine kindly provided by Prof. Federico Marini (University Roma La Sapienza, IT) and adapted by M. Cocchi. ROC-SIMCA analysis was conducted by using a MATLAB routine kindly provided by Dr. Raffaele Vitale (University of Lille, FR). DDSIMCA is available at https://github.com/yzontov/dd-simca (last visited July 2020).

## 3. Results

### 3.1. Authenticity Assessment

The aim was to distinguish grated authentic Parmigiano cheese samples (PR) from “the rest of the world” grated cheese samples, including competitors, sounds-like products, etc. (not-PR). This is a typical one-class model situation, where one-class classifier, such as SIMCA, is the method of choice. The datasets and applied methodology are described in Section 2.4; as for the choice of model complexity, within the framework of alternative-SIMCA, we considered two cases:

(i) only samples of the target class are available, as was indeed our case, since not-PR samples were received at a second time from the Parmigiano Reggiano Cheese Consortium;

(ii) a limited number of non-target class samples are also available and can be considered in the model building phase.

In the first case, only sensitivity can be estimated, and a criterion of choice to estimate the number of components could be selecting the number which allows for achieving the maximum value of sensitivity in cross-validation (Sensitivity_CV). This is an estimate of the predictive capability toward correct acceptance of samples belonging to the target category. A second criterion, as for PCA, is to seek for a minimum in root mean squares error in cross validation (RMSECV). In this case, it is equivalent to considering only the orthogonal distance to the class model to evaluate class belonging, and the focus is on optimizing the number of components so that samples of the target class will have low residuals.

**Figure 3:**
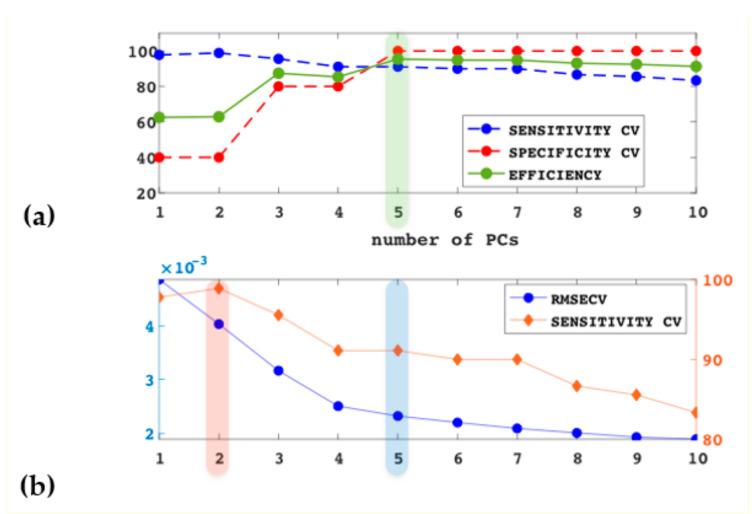
One-class Soft Independent Modeling of Class Analogy (SIMCA) model built using authentic PR samples (PR1st and PR2nd, in Table 1) as a calibration set. (**a**) Sensitivity, specificity and efficiency in CV vs. number of components. Specificity was estimated on five not-PR samples. (**b**) Sensitivity in CV (red line, right *y*-axis) and RMSECV (blue line, left *y*-axis) vs. number of components. The shaded areas in each graph highlights the number of components selected according to each one of the criteria, Sensitivity_CV (red shade), Efficiency_CV (green shade) and RMSECV (blue shade), respectively. Sensitivity in (**a**) and (**b**) are the same.

In Figure 3b are shown the trends of Sensitivity_CV and RMSECV with an increasing number of components; adopting the criterion of maximum value of Sensitivity_CV would lead to the selection of two components as optimal to model the target category. RMSECV does not show a definite minimum, while a monotonous decreasing trend is reached at five components. Thus, the selection of either a four- or five-component model would be recommended in this case. Within the DDSIMCA approach, the nominal significance level is achieved by a two-component model.

In the second case, by using five of the not-PR samples, it was also possible to estimate the Specificity_CV, and thus the criterion of selecting the number of components giving the highest value of efficiency in CV can be adopted. In Figure 3a, the plot of Sensitivity_CV, Specificity_CV and Efficiency_CV values with an increasing number of components is shown. A five-component model corresponds to the highest value of Efficiency_CV. The simultaneous optimization of significance level and number of components by the ROC-SIMCA approach converged to the same number of components (5) and significance level (α = 0.05).

In Table 2 are summarized the classification results corresponding to the different criteria. All models gave good predictions with respect to sensitivity (Sensitivity Test), albeit optimal for a five-PC model. On the other hand, specificity in prediction (Specificity Test) is unsatisfactory when a two-PC model is fit. Thus, with this dataset, it seems that in cases where non-target samples will not be available, the minimum RMSECV can be a more suitable criterion to select the number of components with respect to maximum Sensitivity_CV. DDSIMCA, whose rule is also based on sensitivity (albeit estimated in fit, not in CV), also indicates two PCs as the optimal class dimensionality. However, DDSIMCA is using different statistics to estimate the threshold for the combined distance (*D*) with respect to alternative-SIMCA approach, and showed a higher Specificity Test, i.e., 82% (only two false positive samples); the same result would be obtained by considering four PCs, instead of five, when evaluating the RMSECV trend (Figure 3b).

When samples of the non-target category are available, even considering a few (five in our case), by using Efficiency_CV to estimate the model complexity, five PCs are selected, which leads to the best class model.

**Figure 4:**
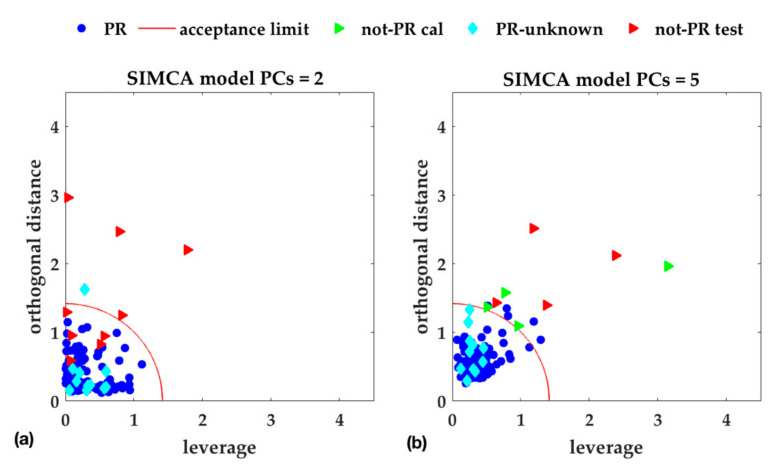
Reduced score distance ($T^{2}$/$T^{2}{}_{lim}$) vs. reduced orthogonal distance (*Q/Q_lim_*), at 95% confidence level. (**a**) One-class SIMCA model with two components, selected according to maximum Sensitivity_CV; (**b**) One-class SIMCA model with five components, selected according to maximum Efficiency_CV (or selected according to minimum RMSECV, i.e., the model is the same). Efficiency_CV has been estimated by the not-PR cal. samples (green triangles). The red circle (radius = 1.414) corresponds to the acceptance class boundary.

To have an idea of sample distribution, Figure 4 shows the reduced *SD* vs. *OD* plot, with the acceptance area, for the two PCs (Figure 4a) and five PCs (Figure 4b) alternative-SIMCA models, respectively. In Figure 4b, the not-PR samples used to assess efficiency are shown by green triangles.

### 3.2. Calibration of Rind Content

**Figure 5:**
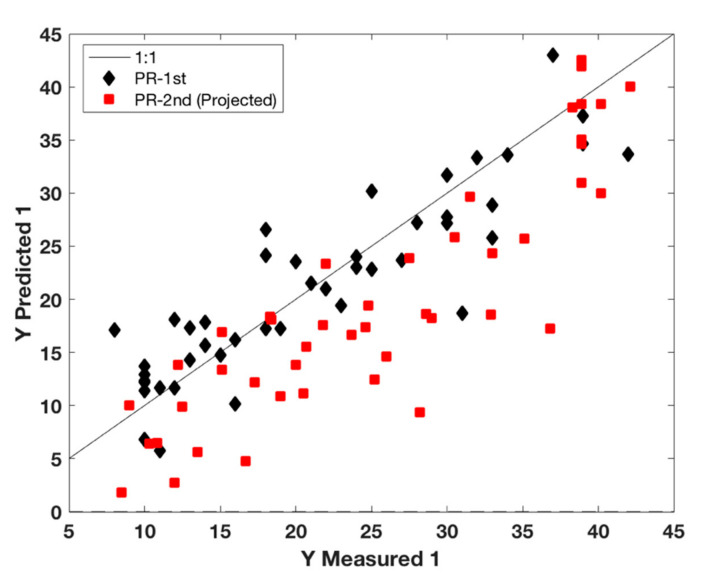
Partial least squares regression (PLS) calibration model for % rind content based on the first sampling set: Measured vs. Predicted rind values. PR 1st samples: black diamonds; PR 2nd set samples: red squares.

For Parmigiano Reggiano grated cheese to be compliant with the protected denomination of origin, it has to be obtained from authentic whole Parmigiano Reggiano cheese wheels and must have a rind content not exceeding 18% (*w*/*w*). To develop the calibration model, we firstly considered the situation closest to what could be a confirmation analysis protocol, i.e., acquiring Raman spectra on intact packaged grated cheese samples and directly estimating the rind content by a calibration model developed beforehand and implemented in the handheld instrument. To this aim, even if the available samples were quite few, we first tested a calibration model developed with the grated cheese samples manufactured in 2015 (the first 40 samples reported as PR 1st in Table 1) and used this model to predict the samples manufactured in 2016 (samples reported as PR 2nd on Table 2), which were also acquired during a different measuring session. The PLS results are shown in Figure 5 and it is evident that there is a systematic error in prediction. This can be somehow expected considering the intrinsic variability between production year, variability due to grinding processes, etc.

Thus, we considered updating the model by augmenting the calibration set including six samples as representative of the PR 2nd set. These were selected by the duplex algorithm and correspond to twelve spectra (one sample was replicated six times).

The results are shown in Table 3. This model shows consistent values of error in fit (RMSEC) and prediction (RMSEP) and has been used to predict the rind content of the unknown-PR samples. When the rind content of these samples become known to us, we could estimate the associated prediction error, i.e., RMSEP is equal to 5.1%, which is consistent and very close to the prediction error associated with the PR 2nd samples not used in calibration.

**Figure 6:**
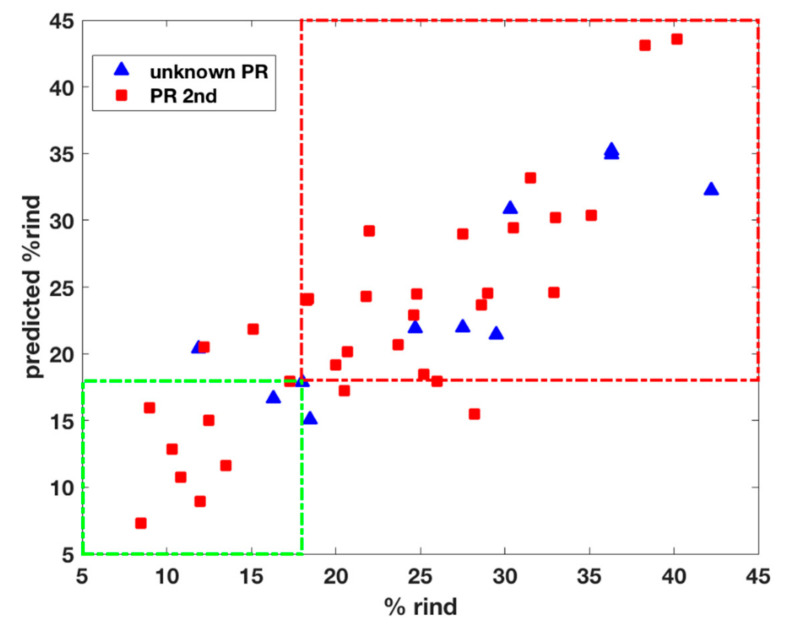
PLS calibration model for % rind content based on the updated calibration set (PR 1st + 6 PR 2nd): only predicted samples are shown, measured vs. predicted rind values. PR 2nd samples (test): red squares; unknown-PR: blue triangles. Samples falling in the green framed area are correctly seen as compliant and samples in the red frame are correctly seen as non-compliant.

In Figure 6, the measured vs. predicted rind content is shown for all samples predicted by the updated calibration model. Even if an RMSEP of 5% is not satisfactory, the variability due to ripening, grinding, etc. has to be considered. However, the model can distinguish compliant from non-compliant PR samples quite well. In fact, only three samples fall in the region of wrongly rejected and three (of which one quite close to the limit) in the region of wrongly accepted, respectively.

**Figure 7:**
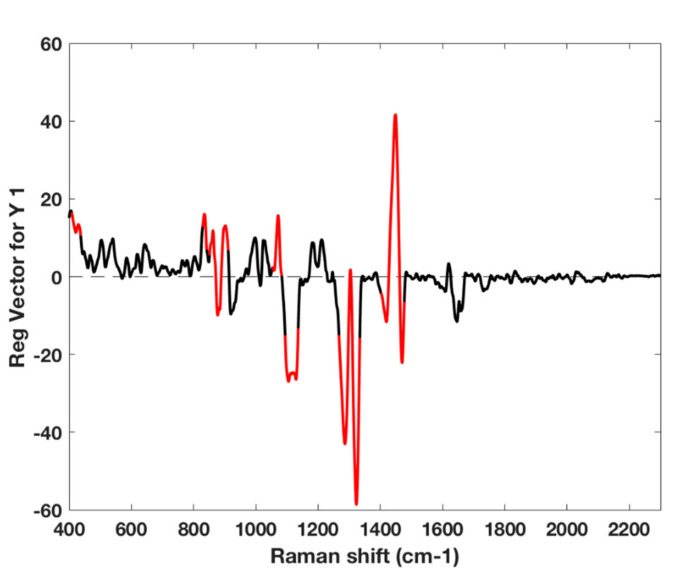
PLS calibration model for % rind content based on the updated calibration set (PR 1st + 6 PR 2nd): regression coefficients with region with variable importance in projection (VIP) scores higher than one highlighted in red.

The relevance of spectral region in the PLS model can be assessed by looking at the regression coefficients together with the variable importance in projection (VIP) [43,44] as variable ranking criteria, as shown in Figure 7. Three main spectral regions are highlighted and are coherent with the chemical composition of cheese and dairy products [13,14,16]:

(i) the spectral range from 1200 to 800 cm^−1^, which can be associated to C-C stretching of aliphatic amino acids (e.g., phenylalanine band at around 1000 cm^−1^, side chains Lys, Asp and Glu around 1065 cm^−1^, Trp at about 1124 cm^−1^), the C-C-N stretching at about 940 cm^−1^, the phosphate stretch around 930 cm^−1^, the C-O ester linkage mode of lipids around 1150 cm^−1^;

(ii) the spectral range from 1400 to 1200 cm^−1^ which can be associated to the amide III band in α-helix structures (1260–1340 cm^−1^),

(iii) the intense band at about 1440 cm^−1^, which can be associated to the CH deformation of fatty acids, carbohydrate and aliphatic amino acids side chains.

Overall, these spectral regions may account for the proteolysis phenomena occurring during ripening and the structural change in the proteins in the rind.

Noteworthily, studies concerning the authentication of Grana Padano cheese [24] reported that the detection of rind addition to grated cheese may be detected by capillary electrophoresis dosing the ratio of the α_s2_ to α_s1_ casein forms, which was found to be higher for the rind; a Raman study focused on the comparison of different phosphorylated casein fractions [45] reported as distinctive for higher phosphorylation, as is the case of α_s2_ casein [46], the Raman bands at 1003, 980, 850, and 830 cm^−1^, which show positive regression coefficients in Figure 7.

**Figure 8:**
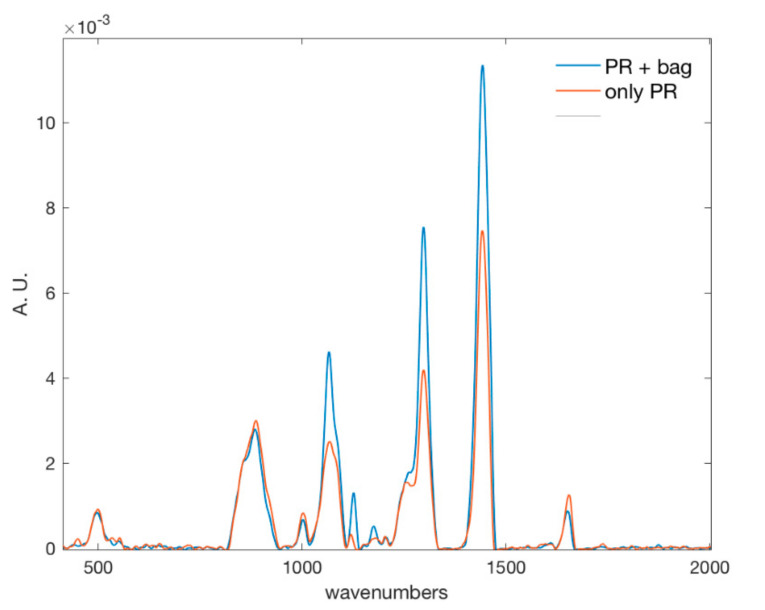
For the same sample the spectrum acquired on grated PR as such (red line) and on packaged grated PR (blue line) are shown. Where the plastic absorbs, the intensity of the blue line is higher than the intensity of the red one.

The salient spectral regions discussed above are consistent with cheese composition; however, while some are specific to cheese, there are also regions which are common to both cheese and packaging material, as can be observed by comparing the spectrum of the same sample acquired as such with the one acquired through the bag (Figure 8). On the other hand, in this feasibility study, the plastic material was the same for each sample. Thus, this contribution affects all samples in the same way. Moving to real application, different materials can be employed in the packaging (plastic type and use of aluminum coupled to plastic) of grated cheese, and consequently more investigation covering the packaging material variability should be carried on testing the robustness of the methodology. Although in commercial products a printed label is also present on the bag, the spectra can be acquired in a region without any printing.

## 4. Discussion

### 4.1. Authenticity Model

The feasibility study presented here and based on handheld Raman device showed very good performance, and all unknown-PR tested sample were correctly predicted.

A strict comparison with other proposed methodologies may be impaired because of the different number of samples (unpacked), different instruments and classification algorithms applied. However, the predictive capability of the different approaches can be generally discussed.

Camin et al. [18] analysed a quite large number of samples (265) and reported a 98% correct classification rate for random forest (RF) classification based on stable light isotope analysis and mineral profiles, which is the reference methodology; the predictive capability of RF model was instead not reported.

Cevoli et al. [20] developed authenticity models based on NIR/MIR spectroscopy, on a calibration set of 146 samples, authentic and compliant, i.e., whose rind content was below 18%, for the PR category and 116 samples for the competitors. The corresponding SIMCA model showed 20% uncorrected classified case, while a model based on artificial neural networks (ANN) based on four classes, namely authentic compliant PR, competitors, defected PR, and authentic non-compliant PR (rind content > 18%), showed a correct test set classification rate of 95%, in the case of the authentic compliant PR category. With respect to these models, we achieved with SIMCA a better predictive performance. Although our dataset included much fewer samples, it covered variability with respect to production year, area of production, period of grating. Moreover, the samples used as test set were not a split of a single sampling.

It is worth underlining that selecting the optimal complexity is crucial in building the SIMCA model, and this is not a trivial task when only samples from the target class are available. Within this specific application, RMSECV proved an effective criterion of choice, but this conclusion cannot be generalized and further investigation comparing several datasets of different typologies is foreseen.

### 4.2. Calibration of Rind Content

The obtained calibration model shows a prediction error for the rind content of about 5%, which, despite being higher with respect to recently proposed models based on NIR [20], i.e., about 3.5%, and NIR imaging [28], i.e., ranging from 2% to 2.5%, may be interesting from the applicative point of view, considering some of the advantages offered by the handheld Raman technique. In fact, there is the possibility to measure directly through packaging without opening it; the measure is not influenced by the varying humidity of the samples, which can be a serious limitation for NIR; and finally, the lower cost and easy handling with respect to NIR-imaging device.

It is worth noting that very few samples of the second set were sufficient to update the calibration model; this is encouraging for the limited effort which can be foreseen for model maintenance. Furthermore, it has to be remarked that this was a feasibility study with a limited number of samples and an improved calibration model can be obtained by enlarging the sampling. In addition, the number of spectra to be acquired on each sample can be increased to better map the packaged sample area.

## 5. Conclusions

Here, we presented a feasibility study to evaluate the potentiality of portable Raman as a fingerprint technique in developing both an authenticity model, by the class modeling approach, of grated Protected Denomination of Origin (PDO) Parmigiano Reggiano cheese and a multivariate calibration model to verify the compliance of authentic grated Parmigiano cheese with respect to the maximum allowed rind content percentage.

The authenticity model’s performance was very good. The calibration model was satisfactory for assessing compliance or not compliance with respect to the threshold of 18% set by regulation, despite the prediction error being quite large, i.e., 5%.

These results, considering that there is room for improvement with a more systematic sampling, are encouraging with respect to the possibility of implementing a methodology based on in situ, fast, non-destructive, quite cheap analytical technique that would enable a much wider screening campaign. In this case, the stable isotopes and elemental analysis, for authentication, and the electrophoretic analysis, for the compliance with respect to rind content, can then be employed as confirmatory analysis.

On the other hand, this represents a small pilot study, which just indicates that the proposed methodology is worthy of further investigation, while not being at all conclusive. In particular, the impact of different packaging materials, including the presence of labels, has to be assessed, as well as a wider sample campaign which needs to be undertaken. At the same time, even if the models are developed in the context of blind analysis for fast screening, a deeper investigation of spectral signatures which may be distinctive for Parmigiano Reggiano would strongly support the application of the approach.

## Figures and Tables

**Table 1:** Summary information on the analyzed grated cheese samples.

Samples Code	% Rind	N°	Production Period	Provenance	Grating-Packaging
PR 1st	8–18	16	September–December 2014	MO(3) MN(1) PR(5) RE(7)	December 2015
PR 1st	19–42	24	September–December 2014	BO(1) MO(2) MN(1) PR(5) RE(3)	December 2015
PR 2nd	8.3–17.5	12	December 2014–April 2015	BO(1) MO(2) MN(1) PR(5) RE(3)	March 2016
PR 2nd	18.3–42	28	January–April 2015	MO(5) MN(1) PR(16) RE(6)	March 2016
PR-unknown	12–42.2	10	December 2014–April 2015	MN(1) PR(6) RE(3)	April 2016
not-PR	-	10	-	Lithuania(5) Germany(3) Estonia(1) Leetonia(1)	-

**Table 2:** SIMCA results (TP = true positive; TN = true negative).

Classification Rule	Criteria to Set n° PC	n° PC	% Sensitivity Fit (TP/N_cal [PR]_)	% Sensitivity CV (TP/N_cal [PR]_)	% Sensitivity Test (TP/N_test_) _[unkonw-PR]_	% Specificity Test (TN/N_test_) _[not-PR]_
D_red_ < sqrt(2) ^1^ α = 0.05	max (Sensitivity CV)	2	100 (90/90)	99 (89/90)	91 (10/11)	55 (6/11)
D_red_ < sqrt(2) α = 0.05	min (RMSECV)	4	95 (86/90)	91 (82/90)	91 (10/11)	82 (9/11)
		5	94 (85/90)	91 (82/90)	100 (11/11)	100 (11/11)
DDSIMCA α = 0.05	Posterior Sensitivity equals to nominal α	2	96 ^2^ (86/90)		100 (11/11)	82 (9/11)
D_red_ < sqrt(2) α = 0.05	max (Efficiency CV) ^3^	5	94 (85/90)	91 (82/90)	100 (11/11)	100 (6/6)
D_red_ < sqrt(2) ROC-SIMCA	Optimize area under ROC in CV	5	94 (85/90)	91 (82/90)	100 (11/11)	100 (6/6)

^1^ T^2^_lim_ = 6.3 (n° PC = 2); T^2^_lim_ = 10.3 (n° PC = 4); T^2^_lim_ = 12.2 (n° PC = 5). ^2^ DDSIMCA, differently from alternative-SIMCA, uses a single χ^2^ reference distribution for the combined distance statistic [40]. ^3^ To estimate Efficiency five samples from not-PR class were used (see Section 2).

**Table 3:** PLS results.

Calibration Set n° Samples (+ n° Replicates)	n° LV	RMSEC	RMSECV	Validation Set n° Samples (+ n° Replicates)	RMSEP
PR 1st: 40 (+4)	3	4.3	5.2	PR 2nd: 40 (+6)	7.6
PR 1st: 40 (+4) PR 2nd: 6 (+6)	3	4.8	5.7	PR 2nd: 34	4.8
				unknown-PR: 10 (+1)	5.1
